# Next-generation sequencing of mitochondrial DNA reveals pathogenic variants and protective haplogroup D4 in esophageal cancer

**DOI:** 10.3389/fgene.2025.1643229

**Published:** 2025-09-22

**Authors:** Xiucheng Jiang, Lan Shi, Mei Zhao, Cui Chen, Tao Tang, Simeng Ji, Bingbing Lv, Lihua Jia, Shuhan Duan, Jinyue Ma, Jiyu Pang, Bo Mu, Yongsheng Zhao, Junbao Yang

**Affiliations:** ^1^ Institute of Basic Medicine and Forensic Medicine, North Sichuan Medical College and Center for Genetics and Prenatal Diagnosis, Affiliated Hospital of North Sichuan Medical College, Nanchong, Sichuan, China; ^2^ School of Laboratory Medicine, North Sichuan Medical College, Nanchong, Sichuan, China; ^3^ Department of Technology and Social Services, Dazhou Vocational College of Chinese Medicine, Dazhou, Sichuan, China; ^4^ Department of Thoracic Surgery, Affiliated Hospital of North Sichuan Medical College, Nanchong, Sichuan, China

**Keywords:** mitochondrial DNA, heteroplasmy, haplogroup, esophageal cancer, variation

## Abstract

**Introduction:**

The germline variations in the mitochondrial genome of esophageal cancer (EC) remain uncertain. Our study aimed to explore the distribution and pathogenicity of mitochondrial genome variations in EC, as well as to identify haplogroups associated with the development of EC.

**Methods:**

We performed next-generation sequencing of the mitochondrial genomes from peripheral blood samples of 146 EC patients and 120 healthy controls. Variant annotation was performed using MitoMap, while pathogenicity prediction was conducted through tools such as MitoTip, SIFT, and PolyPhen2. Moreover, haplogroup classification was carried out using the Haplogrep3 platform.

**Results:**

A total of 1299 mitochondrial variants were identified among 146 EC patients, including 171 novel (previously unreported) mutations. Compared with the healthy control group, the EC cohort exhibited a higher frequency of variants in genes such as ND2, COX1, COX2, 12S rRNA, and 16S rRNA. Three tRNA mutations (7496_T>C, 5771_A>G, and 5613_T>A) were predicted to be potentially pathogenic. Within the protein-coding regions, 14 variants were classified as deleterious based on predictions from 13 independent bioinformatic algorithms. Notably, mitochondrial haplogroup D4 was significantly associated with a decreased risk of developing EC. Furthermore, several mtDNA single-nucleotide polymorphisms (SNPs), including 302_A>AC, 1824_T>C, 1842_A>G, 3010_G>A, 8414_C>T, and 14668_C>T, showed significant associations with EC susceptibility.

**Conclusion:**

We found that the number of variations in multiple regions of the mitochondrial genome in the EC population was higher than that in the control group. Additionally, several potentially pathogenic variants were identified, and haplogroup D4 was suggested as a potentially protective haplogroup against the development of EC.

## 1 Introduction

Mitochondria are essential cytoplasmic organelles responsible for various cellular functions, such as generating energy via oxidative phosphorylation (OXPHOS), regulating apoptosis, maintaining calcium homeostasis, managing lipid metabolism, and mediating metabolic signaling (Shaughnessy et al., 2014). These organelles possess their own mitochondrial genome, which spans 16,569 base pairs and encodes 37 genes-13 of which produce proteins involved in OXPHOS, alongside 22 tRNA genes and 2 rRNA genes (Anderson et al., 1981; Andrews et al., 1999; Rigoli and Caruso, 2014; Taylor and Turnbull, 2005). Additionally, the mitochondrial genome includes a variable sequence called the control region (CR), which harbors the initiation sites necessary for both transcription and replication (Anderson et al., 1981; Taylor and Turnbull, 2005; Stewart and Chinnery, 2015). Individual cells harbor multiple mitochondrial copies, which may present different alleles at the same nucleotide position-a condition known as heteroplasmy (Cavalcant et al., 2019).

Mitochondrial DNA (mtDNA) exhibits heightened vulnerability to carcinogens, resulting in increased susceptibility to damage and mutations-occurring at a frequency 10 to 20 times greater than that observed in nuclear DNA (nDNA) (Liu et al., 2017; Liu et al., 2015). Such alterations can result in mitochondrial dysfunction, potentially triggering cellular deregulation driven by impaired DNA repair mechanisms, thereby contributing to the onset of various diseases, including cancer (Zhuang et al., 2024; Ji et al., 2019). Indeed, numerous studies have demonstrated a link between mtDNA instability and heteroplasmy with different types of cancer (Bonora et al., 2021; Venderbosch et al., 2015; Dahal and Raghavan, 2021; Pérez-Amado et al., 2020; Pérez-Amado et al., 2021; Zhou et al., 2024; Hong et al., 2024).

Esophageal cancer (EC) is one of the most common types of cancer and represents a highly lethal malignancy of the digestive tract with both high incidence and mortality worldwide. Genetic susceptibility is considered one of the key risk factors in the development of EC. According to the Global Cancer Statistics 2020, EC ranks 10th in terms of new cancer cases and sixth in cancer-related deaths globally (Sung et al., 2021). Recent epidemiological data highlight significant geographic variation in its incidence, with the highest rates observed in Asia-particularly in East and South-Central Asia-followed by regions of Africa (Morgan et al., 2022; Abnet et al., 2018). In China, high-incidence areas include northern Sichuan, Henan, Fujian, Guangdong, northern Jiangsu, and Xinjiang (Jia et al., 2024). These regional patterns may be due to genetic background and eating habits of the populations.

Research on germline mtDNA mutations in EC may contribute to uncovering genetic susceptibility mechanisms and exploring potential biomarkers for early screening and personalized prevention. However, compared to studies on somatic mtDNA mutations in EC, research on germline mtDNA variants remains relatively limited (Liu et al., 2017; Ji et al., 2019; Ghadyani et al., 2024). An earlier study collected peripheral blood DNA from esophageal squamous cell carcinoma (ESCC) patients and healthy control subjects in northern India, followed by sequencing of the HVR1 region of mtDNA, through which a significant association between the mtDNA G10398A polymorphism and ESCC was identified, suggesting that this polymorphism may serve as an independent risk factor for the development of ESCC (Darvishi et al., 2007). Nevertheless, the study was limited to sequencing only the HVR1 region rather than the complete mitochondrial genome, which may have led to the omission of certain potentially significant variants. Hence, in northern Sichuan-a region with a particularly high incidence of EC-employing whole mitochondrial genome sequencing in future investigations would provide a more comprehensive characterization of the mitochondrial genetic landscape underlying esophageal carcinogenesis in this population.

Germline variations in mtDNA are commonly classified into haplogroups, which are distinguished by particular sets of mtDNA mutations and represent specific geographic origins and ancestral populations (Yuan et al., 2021). Recent studies have confirmed that these mtDNA haplogroups are linked to the susceptibility to several types of cancer (Li et al., 2018; Cocoş et al., 2018; González et al., 2021; Yan et al., 2023). Moreover, Specific mtDNA single-nucleotide polymorphisms (SNPs) have also been associated with an increased risk of cancers (Yuan et al., 2021). Early studies have identified haplogroups D4 and D5 as potential susceptibility markers for EC in two high-incidence regions of China: the Chaoshan area and the Taihang Mountain area (Li et al., 2011; Li et al., 2007). Nevertheless, mtDNA haplogroups show substantial diversity across ethnic populations from different geographical locations. To date, it still remains inconclusive whether mtDNA haplogroups or SNPs constitute significant risk factors for EC within the population in northern Sichuan, China.

The aim of this study was to explore the distribution and pathogenicity of mitochondrial genome variations in EC, as well as to identify haplogroups associated with the development of EC in northern Sichuan, China. To this end, we collected peripheral blood samples from 146 patients with esophageal cancer and 120 healthy individuals, extracted mtDNA, and performed next-generation sequencing. Variant annotation and pathogenicity prediction were conducted using tools including MitoMap, MitoTip, and SIFT, while haplogroup classification and analysis of their distribution across different groups were carried out using HaploGrep3.

## 2 Materials and methods

### 2.1 Sample collection and DNA extraction

Participants were recruited from two cohorts: individuals diagnosed with EC and healthy controls, all coming from a high-incidence EC area in northern Sichuan, China. The EC group comprised patients who sought treatment at the Affiliated Hospital of North Sichuan Medical College between June 2022 and November 2024. The control cohort included individuals with nonneoplastic diseases who also visited the same hospital within the same timeframe and lived in the same area as the EC group. EC diagnoses were verified via pathological tissue examination. In total, the study included 146 EC patients and 120 healthy controls from the Chinese population.

Peripheral blood samples were obtained from both groups using EDTA anticoagulant tubes (Shanghai, BD). Genomic DNA was Extraction from all samples utilizing the Tiangen Blood Genomic DNA Extraction Kit (centrifugal column type; Tiangen, China). The extracted DNA was kept at −80 °C until further use. This study was approved by the Medical Ethics Committees of North Sichuan Medical College (NSMC [2022] 08 and NSMC [2024] 026). Participants were from non-consanguineous families and included individuals whose parents and grandparents were native residents who had lived in the sampling region for at least three successive generations. Informed consent was obtained from all subjects, in accordance with the principles outlined in the Declaration of Helsinki (World Medical Association Declaration of Helsinki, 2001).

### 2.2 mtDNA amplification, template preparation, and sequencing

The library preparation was performed using the RealCap^®^ Human ChrMT Kit (Homgen Biotechnology Company, Shanghai, China) following the manufacturer’s protocol. Initially, target mtDNA regions were amplified using multiplex polymerase chain reaction amplification. Each 30 μL reaction system contained 5 μL Human ChrMT MIX, 10 μL of 3× EnzymeHF, 20 ng of genomic DNA, and 15 μL of nuclease-free H_2_O. PCR amplification conditions were as follows: an initial denaturation at 98 °C for 3 min; 13 cycles of 98 °C for 20 s and 58 °C for 4 min; followed by 7 cycles of 98 °C for 20 s and 72 °C for 1 min; and a final extension at 72 °C for 2 min, then held at 10 °C. The amplified products were purified using Homgen DNA Clean Beads. Subsequently, indexes were added through a second round of PCR to enrich the target regions. The index-adding reaction was also conducted in a 30 μL system, composed of 18 μL purified PCR product, 10 μL of 3× EnzymeHF, 1 μL of I5-TS_XXX, and 1 μL of I7-MPI-XXX. The thermal cycling conditions included an initial step at 98 °C for 2 min, followed by 6 cycles of 98 °C for 15 s, 58 °C for 15 s, and 72 °C for 30 s, with a final extension at 72 °C for 2 min, and a hold at 10 °C. The reamplified products were again purified using Homgen DNA Clean Beads to obtain the final enriched library. The DNA library products were quantified using a Qubit fluorometer and quality-checked with the Agilent 2,100 Bioanalyzer. After quantification, the qualified libraries were sequenced on the Illumina Novaseq6000 using paired-end 150 bp sequencing.

### 2.3 Sequencing data processing

To ensure the reliability of downstream analyses, low-quality reads in the mitochondrial sequencing data were filtered using fastp v0.23.4 (Chen et al., 2018). Subsequently, the cleaned FASTQ files were aligned to the human mtDNA reference sequence (rCRS, GenBank accession number NC_012920) (Andrews et al., 1999) using BWA v0.7.17 (Li and Durbin, 2010), and the resulting alignments were saved in BAM format. Variant sites were subsequently detected from the BAM files using SAMtools v1.9 (http://samtools.sourceforge.net/) and exported in variant call format (VCF). Finally, consistent sequence FASTA files were generated with BCFtools v1.18 (https://samtools.github.io/bcftools/). Default parameters were used for all bioinformatic programs.

### 2.4 Variant annotation and pathogenicity prediction

All mtDNA variants extracted from raw VCF files were annotated using Mitomap, a human mitochondrial genome database (http://www.mitomap.org), including region (protein coding, tRNA, rRNA or non-coding), mutation type (transition, transversion, insertion or deletion), effect on amino acid change (nonsynonymous or synonymous) and patient report related to variations. Variations not recorded in the Mitomap database were regarded as novel.

Inter-species conservation of altered nucleotides or amino acids was assessed utilizing mitochondrial genome sequences from 44 primates (Supplementary Table S1). The conservation index (CI) was defined as the proportion of species possessing the wild-type nucleotide or amino acid, determined by comparing the human nucleotide or amino acid with those of the other 43 species. A higher CI indicates greater evolutionary constraint, thereby implying an increased likelihood of functional and pathogenic significance for the variant. Variants with potential pathogenic effects were selected according to the following criteria (Shaughnessy et al., 2014): a population frequency of less than 1% among 120 healthy controls-those exceeding this threshold were considered polymorphisms; and (Anderson et al., 1981) high evolutionary conservation of the altered nucleotides or amino-acids (CI > 75%), suggesting a probable impact on molecular function.

The variants identified in 22 tRNAs were assessed for pathogenicity using the MitoTIP (Sonney et al., 2017): >16.25 (Quartile: 75%–100%) = Likely Pathogenic (LP); 12.66–16.25 (Quartile: 50%–75%) = Possibly Pathogenic (PP); 8.44–12.66 (Quartile: 25%–50%) = Possibly benign (PB); and <8.44 (Quartile: 0%–25%) = Likely Benign (LB). LP and PP were considered deleterious in this study. Mitochondrial tRNA secondary structures were predicted using tRNAscan-SE 2.0 (Lowe and Chan, 2016) and schematically visualized with VARNA v3-93 (Darty et al., 2009). Moreover, A total of 13 bioinformatic programs were employed to assess potentially pathogenic variations in the protein-coding region including PolyPhen2 (http://genetics.bwh.harvard.edu/pph2), SIFT (http://sift.bii.a-star.edu.sg), VEST (http://www.cravat.us), Mitoclass.1 (https://github.com/tonomartin2/MITOCLASS.1/), SNPdryad (http://snps.ccbr.utoronto.ca:8080/SNPdryad/), AlphaMissense (https://alphamissense.hegelab.org/), CADD (http://cadd.gs.washington.edu), PROVEAN (http://provean.jcvi.org), Mutation Assessor (http://mutationassessor.org), EFIN (http://paed.hku.hk/efin) and MLC. The classification of pathogenicity and corresponding scores for each prediction tool are detailed in Supplementary Table S2. All of the bioinformatic programs are freely available in MitImpact (Castellana et al., 2015). A high probability of being pathogenic for mitochondrial function and association with EC was attributed to variations predicted as deleterious by more than six of the 13 programs.

### 2.5 Determining heteroplasmic and homoplasmic variants

We calculated the heteroplasmic frequency (HF, %) by dividing the number of reads supporting the variant by the total reads at each mitochondrial genome site, thereby determining the proportion of variant alleles. To minimize false-positive results, variants with an HF below 1% were excluded. Variants with an HF between 1% and 98% were classified as heteroplasmic, while those with an HF equal to or exceeding 98% were defined as homoplasmic.

### 2.6 mtDNA haplogroups and SNPs

The obtained VCF files were processed using the Haplogrep3 platform (https://haplogrep.i-med.ac.at/) to classify mtDNA haplogroups. Macro- and micro-haplogroups were annotated according to PhyloTree 17 (www.phylotree.org). Variants detected in comparison to the reference genome were designated as mtDNA SNPs. To enhance analytical rigor, SNPs with a minor allele frequency (MAF) below 5% in both cases and controls were excluded from subsequent analyses.

### 2.7 Population comparisons

To investigate the genetic relationships between people from high-incidence regions of EC in northern Sichuan and other high-risk areas in China, we obtained 119 mtDNA sequences from Chaoshan area, one of high-incidence regions of EC in China and 9,209 mitochondrial genomes from 21 populations across various provinces in China (Li et al., 2007; Li et al., 2019). Detailed information on all reference populations is provided in Supplementary Table S3.

In order to obtain a deeper understanding of population relationships across different group sets, principal component analysis (PCA) based on haplogroup frequencies was conducted using MVSP 3.22 software (Kovach, 1999), and the results were visualized with the “ggplot2″ package in R software (https://www.r-project.org).

### 2.8 Statistical analyses

Statistical analyses were conducted using Python (https://www.python.org), and the following tests were applied for the different analyses: Mann-Whitney U Test, Chi-Square Test, Fisher’s Exact Test and Logistic Regression. Statistical power was also assessed using Python. R packages “circlize”, “ggplot2” and “cellranger” were used for graphic representations. In all analyses, statistical significance was defined as a P-value of <0.05.

## 3 Results

### 3.1 Distribution of mtDNA variations

**FIGURE 1:**
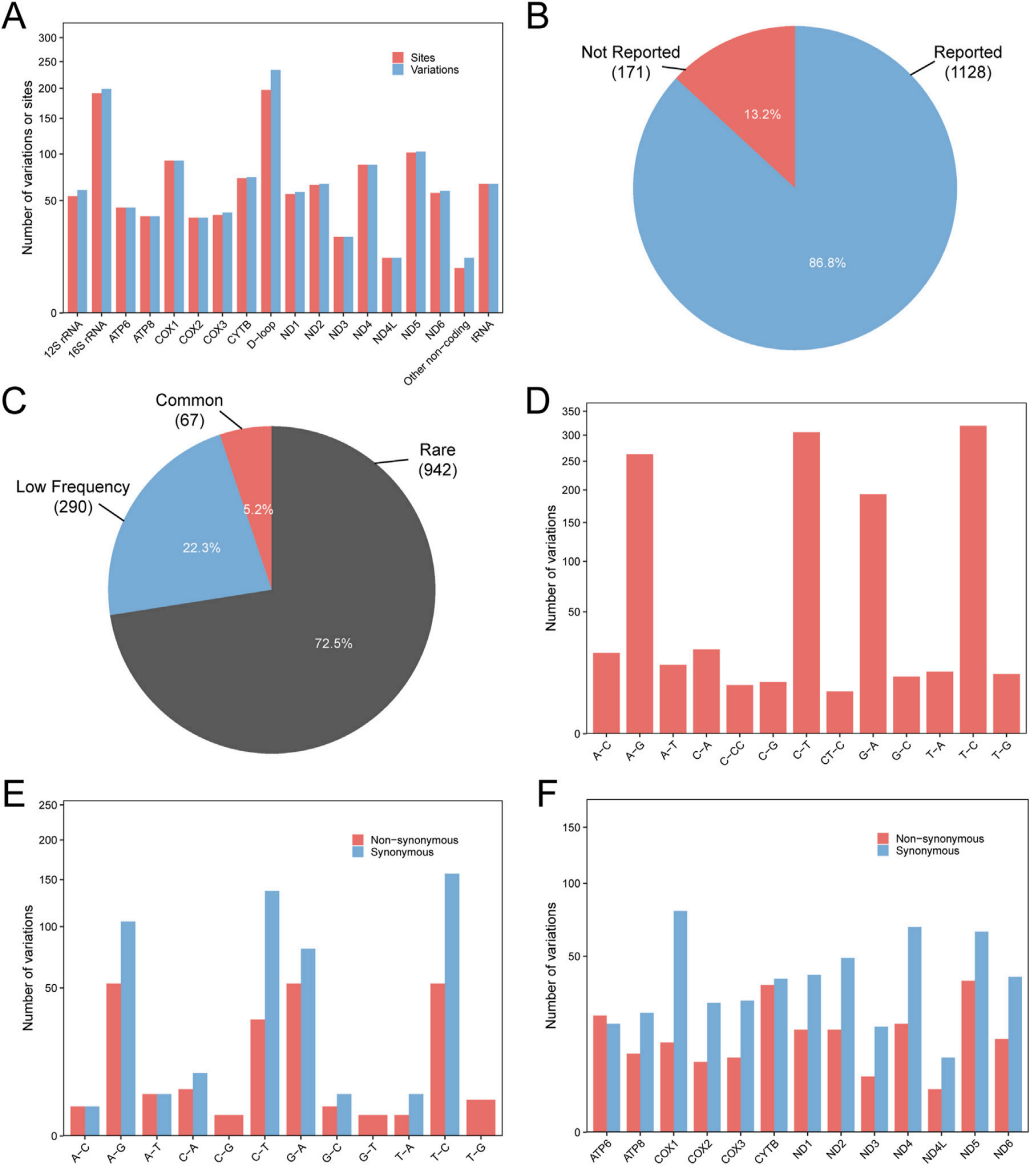
Characterization of 1299 mtDNA variations. **(A)** Distribution of mtDNA variations in the entire mitochondrial genome. **(B)** The proportion of variations that were reported or not reported in MitoMap database. **(C)** Variants were grouped based on Allele Frequency (AF) in MitoMap. Common variants: AF ≥ 5%; low-frequency variants: 0.5% ≤ AF < 5%; rare variants: AF < 0.5%. The percentage indicates the proportion of each group. **(D)** Distribution of different types of variation. **(E)** The proportion of synonymous versus nonsynonymous variations across different types of substitutions. **(F)** The proportion of synonymous and nonsynonymous variations in protein-coding region.

A total of 1299 variations in 1234 sites were identified in 146 EC cases, and the mtDNA control region (D-loop) and rRNA regions exhibit a higher number of variants compared to other genomic regions (Figure 1A). A cohort of 146 EC patients harbored 171 novel (previously unreported) genetic variations, comprising 19 non-synonymous substitutions and 10 frameshift mutations (Figure 1B). Rare variants (942/1299, 72.5%) accounted for the largest proportion among all variant types (Figure 1C). Furthermore, among these 1299 variants, 108 have been previously documented to associate with diverse pathologies, including melanoma, Alzheimer’s disease (AD), and related disorders (Supplementary Table S4). Single-base substitution (especially T>C, C>T, A>G and G>A) was the main component of mtDNA variations (Figure 1D). About 28% (190/669) transitions and 45% (19/42) transversions were non-synonymous, suggesting that transversion was more likely to alter the encoded amino-acid and affect the structure or function of protein than transition did (Figure 1E). In all protein-coding regions, with the exception of the ATPase6 region where non-synonymous mutations (n = 22) outnumber synonymous mutations (n = 19), synonymous mutations predominate to varying extents across the remaining regions. In addition, ATPase6 (22/41, 53.66%), CYTB (35/73, 47.95%), and ND5 (37/102, 36.27%) genes harbored relatively high ratio of nonsynonymous variation (Figure 1F).

**FIGURE 2:**
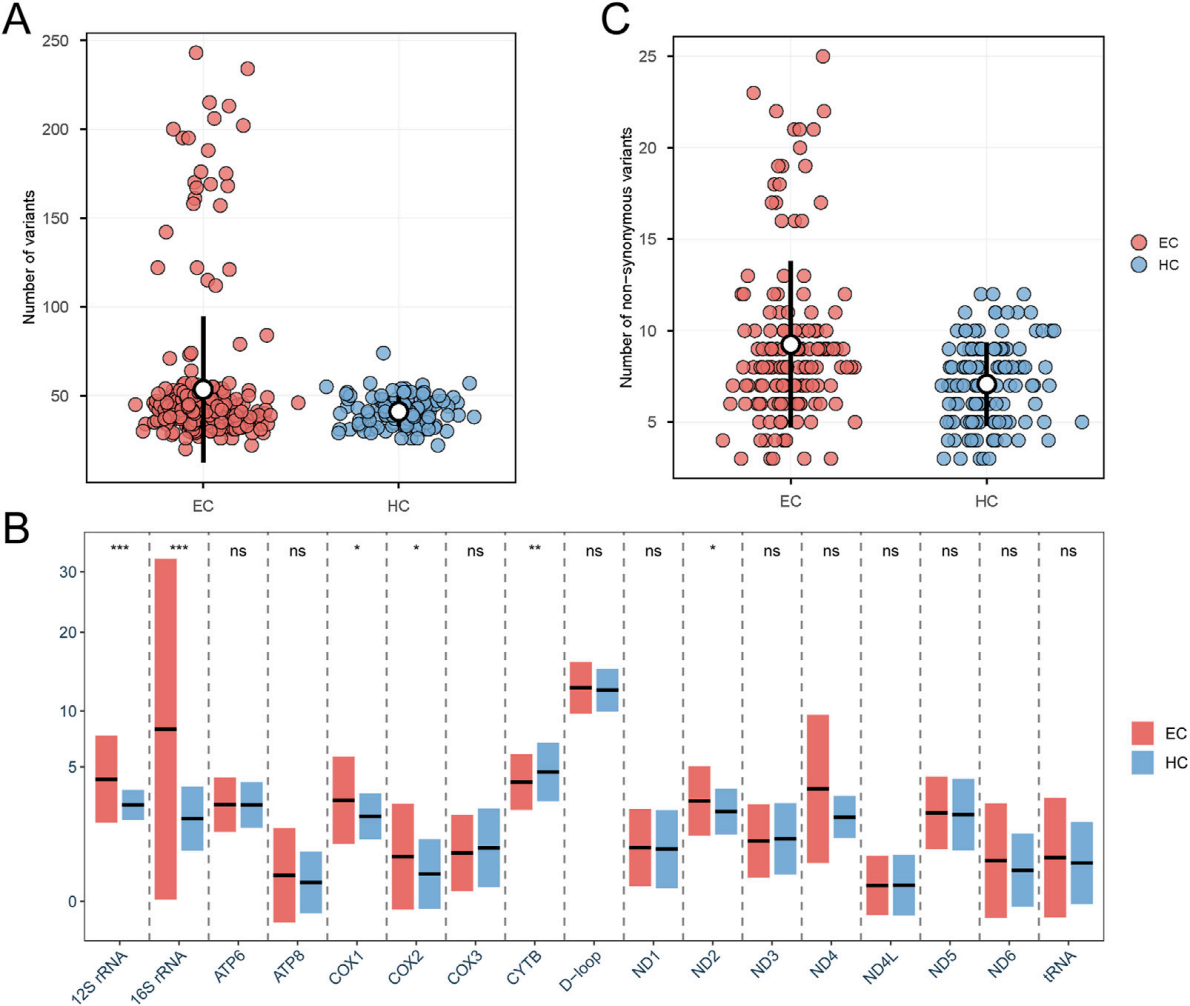
Variation distribution differences between EC and healthy control group. **(A)** The distribution of the number of variants per individual in both groups. **(B)** The distribution of variant counts across different genomic regions for each sample in both groups. The black horizontal lines indicate the mean number of variants for each region. The paired rectangles show the mean ± standard deviation (SD) of variants for EC (coral) and HC (light blue). ^∗∗∗^P < 0.001; ^∗∗^P < 0.01; ^∗^P < 0.05; P-values for Mann-Whitney U test. ns, not significant. EC, Esophageal Cancer. HC, Healthy Controls. **(C)** The average number of variants in the coding regions for both groups.

**FIGURE 3:**
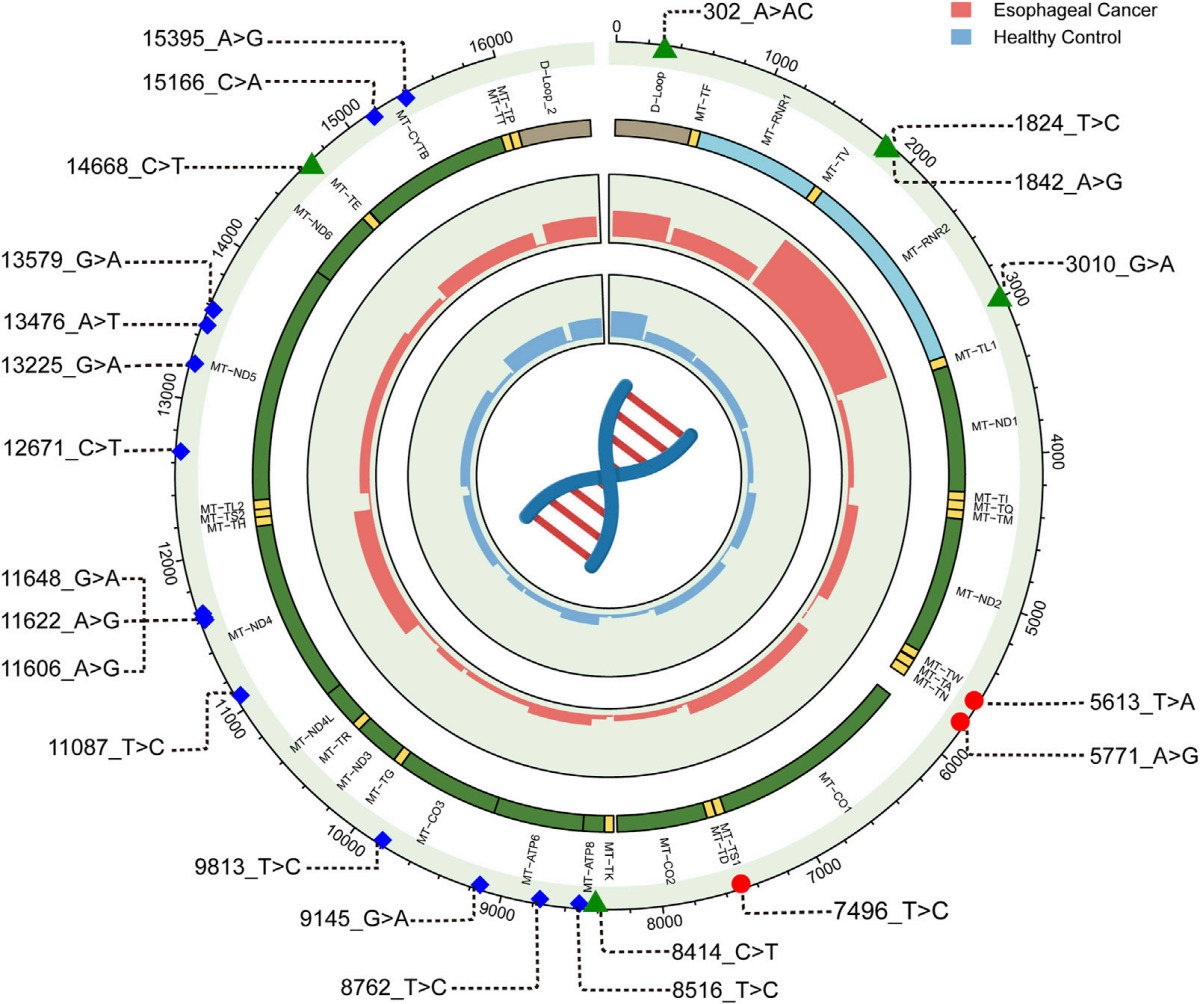
The distribution of mutations in EC and healthy control group. Outer Circle: The outer circle shows the average number of mutations per region for each individual in the EC group. Inner Circle: The inner circle displays the average number of mutations per region for each individual in the healthy control group. Red dots represent mutations predicted by MitoTip to be possibly pathogenic, blue diamonds indicate mutations in the protein-coding regions predicted as deleterious by more than half of the bioinformatic programs, and green triangles denote SNPs that are present in both the EC group and the healthy control group, with a significant difference in their distribution.

Additionally, we analyzed the distribution of the number of variants per individual in two groups and found that the average number of variants per individual was higher in the EC group (mean = 53.5) than in the healthy control group (mean = 41.07), although the Mann-Whitney U test result showed no significant difference (P = 0.2383) (Figure 2A). We then examined the distribution of variant counts across different genomic regions for each sample. In this analysis, we observed a statistically significant difference between two groups, with EC group presenting more variants in ND2, COX1, COX2, 12S rRNA and 16S rRNA (Figures 2B, 3). Furthermore, we assessed the average number of variants in the coding regions for both groups and found that the EC group (mean = 9.25) had a higher average number of variants in the coding regions compared to the healthy control group (mean = 7.08) (Mann-Whitney U test, P = 1.55E−4; Figure 2C).

### 3.2 The level of heteroplasmy in mtDNA variations

**FIGURE 4:**
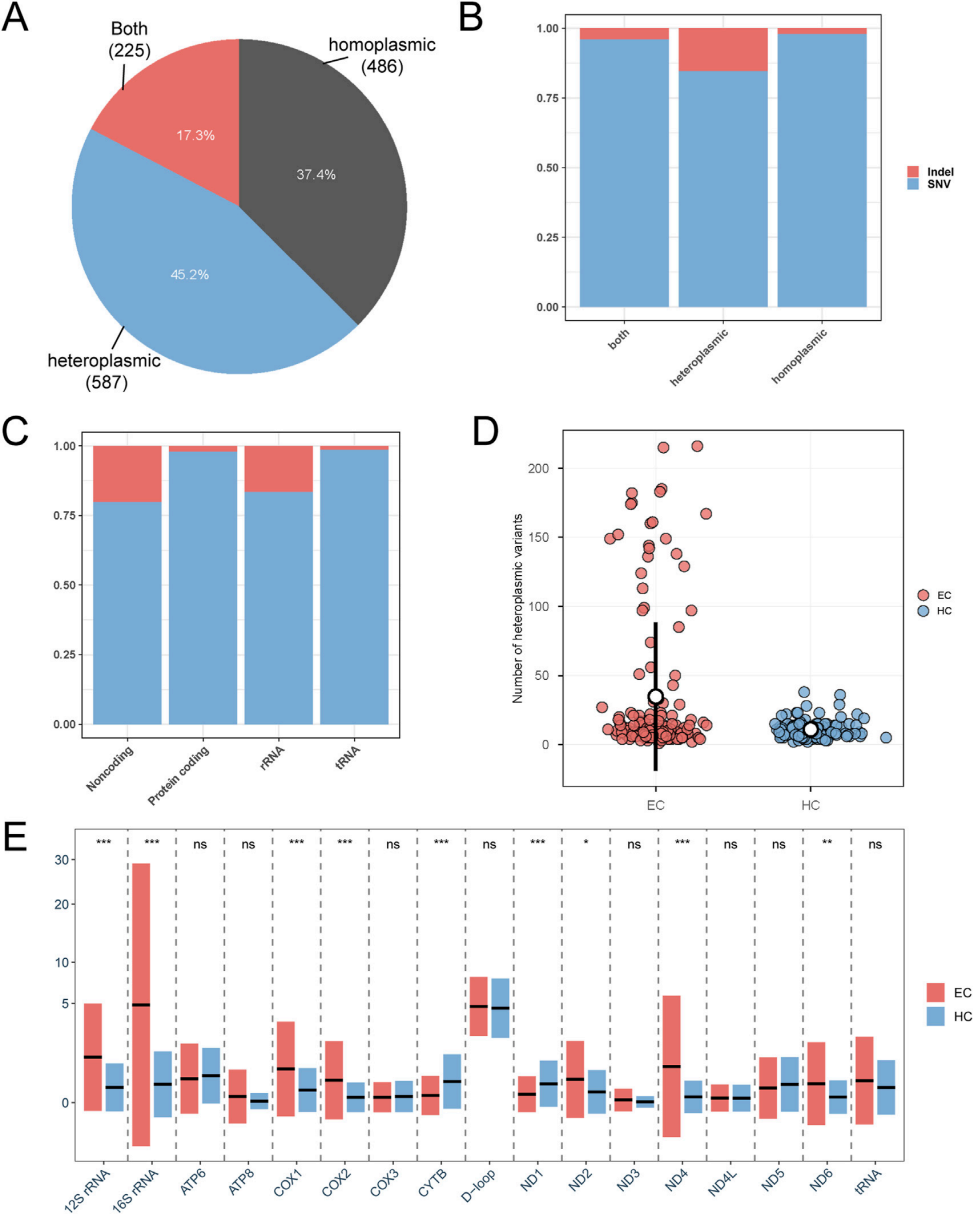
The distribution of heteroplasmic mtDNA variations. **(A)** Proportion of variants present only in the heteroplasmic or the homoplasmic state or present in both. Present in both indicates that some individuals exhibit the heteroplasmic state while others show the homoplasmic state for the same variant. **(B,C)** Distribution characteristics of indels in the mitochondrial genome. **(D)** The distribution of the number of heteroplasmic variants per individual in EC and healthy control group. **(E)** The distribution of heteroplasmic variant counts across different genomic regions for each sample in both groups. The black horizontal lines indicate the mean number of heteroplasmic variants for each region. The paired rectangles show the mean ± standard deviation (SD) of heteroplasmic variants for EC (coral) and HC (light blue). ^∗∗∗^P < 0.001; ^∗∗^P < 0.01; ^∗^P < 0.05; P-values for Mann-Whitney U test. ns, not significant. EC, Esophageal Cancer. HC, Healthy Controls.

To mitigate the potential contamination from nuclear mtDNA segments (nuMTs), only sequencing reads that uniquely aligned to the mitochondrial genome were included in subsequent analyses. These analyses encompassed both homoplasmic variants (HF ≥ 98%) and heteroplasmic variants (1% ≤ HF < 98%). A total of 1298 mtDNA variants were identified (Supplementary Table S5), with 486 (37.4%) classified as homoplasmic, 587 (45.2%) as heteroplasmic, and 225 (17.3%) observed in “both” states (i.e., homoplasmic in some individuals and heteroplasmic in others; Figure 4A).

Of the 1298 mtDNA variants analyzed (the remaining one variant was excluded due to their HF being below 1%), single nucleotide variants predominated (SNVs; 1189, 91.53%) while insertions/deletions accounted for 8.47%. Notably, distinct distribution patterns emerged between variant types: Indel events demonstrated preferential occurrence characteristics when comparing heteroplasmic and homoplasmic variants. Specifically, heteroplasmic mutations exhibited significantly higher indel representation relative to their homoplasmic counterparts (Chi-Square Test, P = 6.43e-18, Fisher’s Exact Test, P = 1.075e-17; Figure 4B). These findings align with prior research demonstrating that heteroplasmic mutations exhibit a greater propensity to accrue deleterious genetic alterations compared to their homoplasmic counterparts (Ding et al., 2015; Wang et al., 2022). In addition, our analyses further identified regional disparities in mitochondrial genome indel distribution (Chi-Square Test, P = 1.21e-43, Fisher’s Exact Test, P = 1.00e-05, Figure 4C). Coding regions displayed markedly lower indel frequencies (n = 15), contrasting with predominant concentrations in non-coding (n = 50) and rRNA-containing segments (n = 43). This spatial heteroplasmy aligns with established mechanisms favoring deleterious variant retention in non-coding domains, where selective constraints are reduced (Figure 4C).

We also explored the distribution of variant counts per individual in both groups and observed that the EC group had a higher average number of variants (mean = 34.78) than the healthy control group (mean = 10.97) (Mann-Whitney U test, P = 0.0394) (Figure 4D). Additionally, we assessed the variant distribution across various genomic regions and found a statistically significant difference, with the EC group exhibiting more variants in ND2, ND4, ND6, COX1, COX2, 12S rRNA, and 16S rRNA regions (Figure 4E).

### 3.3 Pathogenicity prediction of mtDNA variations

**FIGURE 5:**
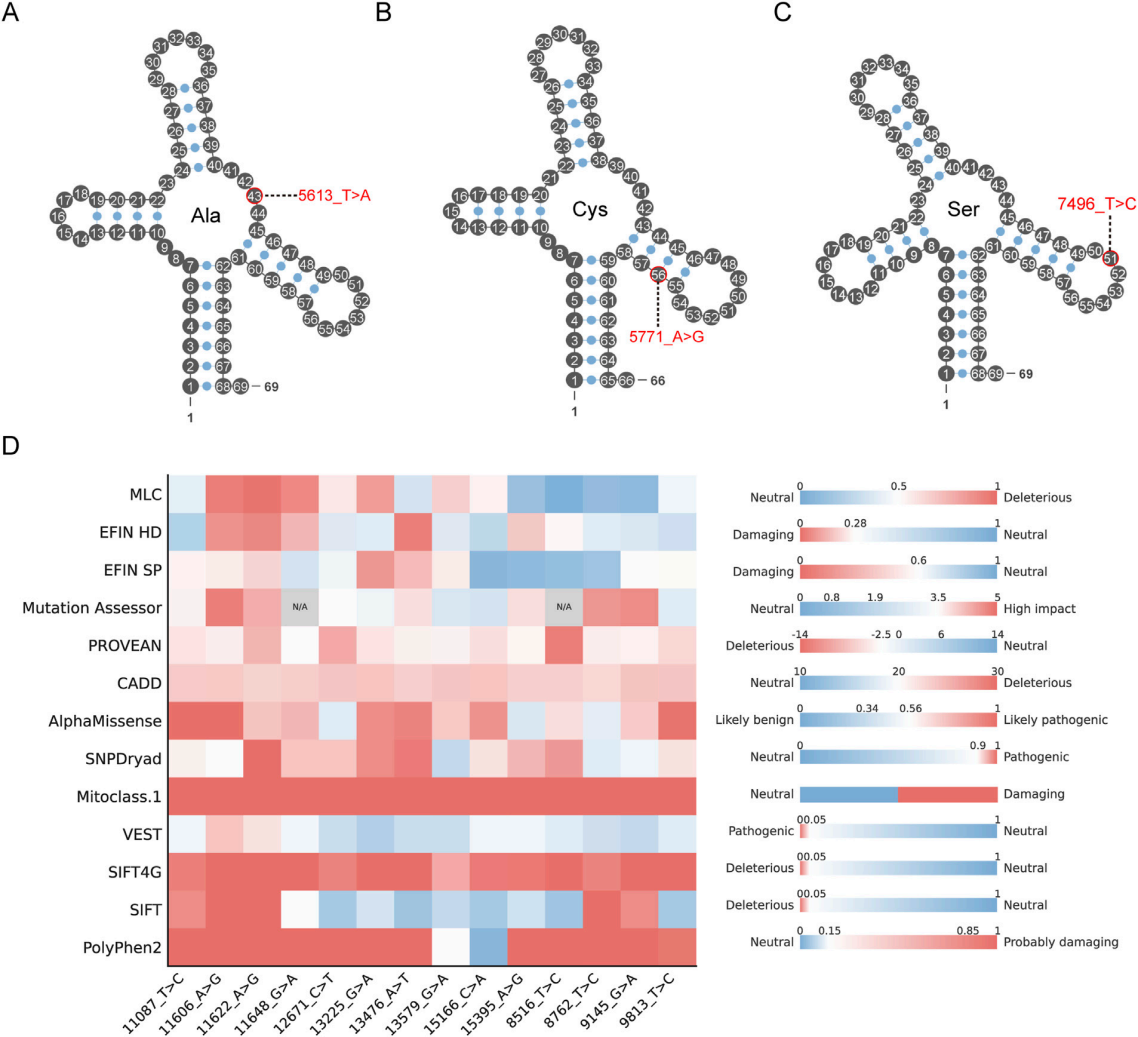
Pathogenicity prediction of variations. **(A–C)** Possibly pathogenic tRNA variations in EC Schematic structures of 3 mitochondrial tRNAs are shown. Dashed line points out the position of tRNA variation. **(D)** Distribution of pathogenicity prediction for variants in protein-coding regions by 13 bioinformatic programs. Each bioinformatic program is accompanied by a color scale on the right, where the minimum and maximum scores are indicated above each bar. Mitoclass.1 is the only bioinformatic program that outputs categorical results. Variants without results predicted by the tool are marked as N/A.

In the RNA coding region, 60, 199 and 67 variations were identified in 12S rRNA, 16S rRNA and tRNAs, respectively. Based on their frequencies in control group and conservation of the altered nucleotides, 14 variants in the tRNA region were included in the analysis. Among these, the 7575_T>C variant was detected at a frequency of 16 in the EC cohort, whereas it was absent in the control group. Furthermore, 6 of these 14 variants have previously been reported to be associated with diseases such as hearing loss and coronary heart disease (7496_T>C, 15901_A>G, 5514_A>G, 4395_A>G, 15910_C>T and 5592_A>G). Following pathogenicity prediction using MitoTip, three variants-5613_T>A, 5771_A>G, and 7496_T>C-were classified as possibly pathogenic. These variants are located within tRNA-Ala, tRNA-Cys, and tRNA-Ser, respectively, with corresponding MitoTip scores of 59.30%, 54.60%, and 58.30% (Supplementary Table S6; Figures 3, 5A–C). A total of 502 synonymous, 212 non-synonymous and 10 frameshift variations were detected in protein-coding region. Among the 212 non-synonymous alterations, 55 variations were considered as potentially pathogenic according to their frequencies in control group and conservation of the altered amino-acid. These 55 selected variations were further assessed by 13 bioinformatic programs, and 14 of them were predicted as deleterious by more than half of the programs (Supplementary Table S7; Figures 3, 5D). Interestingly, among these 14 potentially pathogenic variants, 11606_A>G, 11622_A>G, and 11648_G>A were observed with a frequency of 18 in the EC group, while their frequency was 0 in the healthy control group. Notably, 11622_A>G was predicted to be a pathogenic variant by all prediction programs, suggesting that it may represent an important genetic marker in the development of EC.

### 3.4 Association between mtDNA haplogroups and SNPs and EC risk in northern Sichuan population

To investigate the association between genetic mtDNA variation and EC risk, mtDNA haplogroups were annotated in 146 EC cases and 120 healthy controls form Northern Sichuan, China. As shown in Supplementary Table S8, patients were categorized into nine major haplogroups. Among them, haplogroup F was the most prevalent clade in EC patients and haplogroup D4 was the most prevalent clade in healthy controls (29 cases [19.86%] and 23 controls [19.17%]).

Compared to other haplogroups, haplogroup D4 was significantly less prevalent in EC patients (9.59%, n = 14) than in healthy controls (19.17%, n = 23), indicating a markedly lower risk of EC (OR 0.447, [95% CI 0.219–0.914], P = 0.032) (Table 1). However, no significant association was detected when haplogroup B and F were used as the reference. A possible explanation for this outcome is that OR and p-value calculations rely on comparisons with the reference group. The risk difference between haplogroups D4 and B or F may be smaller than that between D4 and other reference groups, leading to reduced statistical power (Supplementary Table S9).

**TABLE 1:** Association between mtDNA haplogroups and EC risk with other haplogroups as reference group.

Haplogroup	EC	Healthy controls	OR (95% CI)	P-value
A	14	5	2.439 (0.853–6.980)	0.098894417
B	25	16	1.343 (0.680–2.651)	0.495298461
D4	14	23	0.447 (0.219–0.914)	**0.032058937**
D5	7	2	2.971 (0.606–14.578)	0.191219544
F	29	22	1.104 (0.596–2.044)	0.875773212
G	12	6	1.701 (0.619–4.678)	0.336299637
M7	15	10	1.260 (0.544–2.916)	0.675372925
R	14	16	0.689 (0.322–1.477)	0.436366131
Z	3	7	0.339 (0.086–1.339)	0.192992499
Others	13	13	0.805 (0.358–1.808)	0.679823462

Bold entries indicate statistical significance. OR, odds ratio; CI, confidence interval.

**FIGURE 6:**
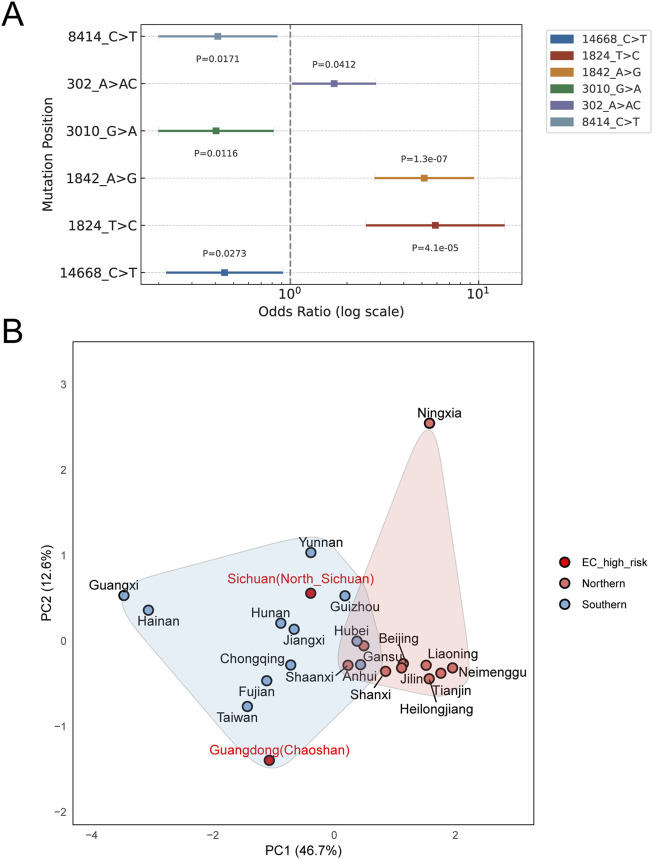
**(A)** Association between mtDNA SNPs and the risk of EC. Using logistic regression to analyze the correlation between SNPs and EC. The abscissa represents the odds ratio (OR) value and 95% confidence interval (CI) in log scale, and the ordinate represents different significant SNPs. **(B)** PCA plot showing the genetic relationships between people from high-incidence regions of EC in northern Sichuan and other 22 populations from different provinces of China based on the haplogroup frequencies.

To clarify the association between mtDNA SNPs and EC risk, we screened for common SNPs with allele frequencies higher than 5% in our cohorts (Supplementary Table S10). Using this criterion, 101 SNPs were identified in control cohort. As shown in Figure 6A, six SNPs showed significant associations with EC risk in our control cohort. In our cohorts, six SNPs were found to be significantly associated with the risk of EC (Figures 3, 6A). Among them, 8414_C>T and 14668_C>T are D4-specific SNPs, further supporting their stable association with the reduced risk of EC. Thus, the association of haplogroup D4 specific SNPs with EC provides further evidence that haplogroup D4 is associated with reduced EC risk in the Northern Sichuan Chinese population.

### 3.5 Genetic relationship between Northern Sichuan EC high-incidence people with other populations

We conducted a PCA using dimensionality reduction to uncover the underlying structure in the complex data. The first two principal components explained 59.3% of the total variance. We found that the populations primarily cluster according to geographic regions. The population from the northern Sichuan EC high-incidence area shared a closer genetic relationship with the populations from Yunnan and Guizhou (Southwestern China), while the population from the Chaoshan EC high-incidence area was more genetically similar to those from Fujian and Taiwan (Southeastern China). Therefore, no close genetic clustering was observed between the populations from the northern Sichuan and Chaoshan areas (Figure 6B).

## 4 Discussion

This study employed next-generation sequencing to sequence the mitochondrial genomes of individuals with EC and healthy controls from the high-incidence region of northern Sichuan, with the aim of elucidating the association between mitochondrial genetics and the incidence of EC. Our study found that the mtDNA control region (D-loop) and rRNA regions exhibit a higher number of variants compared to other genomic regions in EC group. The D-loop and rRNA regions are considered the two major hotspots for mutations in the mitochondrial genome (World Medical Association Declaration of Helsinki, 2001; Masuda et al., 2012). Previous studies have comprehensively characterized mtDNA in EC and found that mutations in the D-loop region occur at a high frequency. These mutations are associated with changes in mtDNA content and are closely linked to processes such as tumor energy metabolism and apoptosis (Zhuang et al., 2024). In addition, earlier research identified mutations in the rRNA regions, suggesting that these regions may play a significant role in the development of EC (Masuda et al., 2012). A study utilizing public database resources identified 52 extremely rare rRNA mutations and, employing a proprietary mt-rRNA pathogenicity scoring system, evaluated candidate pathogenic variants, further demonstrating that mutations in mitochondrial rRNA are closely linked to human disease (Smith et al., 2014). We also observed a statistically significant difference between two groups, with EC group presenting more variants in ND2, COX1, COX2, 12S rRNA and 16S rRNA. This result is analogous to a recently published study on mitochondrial germline mutations in idiopathic pulmonary fibrosis (IPF), which reported that the average number of COX3 non-synonymous variants per patient was significantly higher in the IPF group than in controls (Lee et al., 2025). Protein-coding genes and rRNAs are essential for OXPHOS (Kim et al., 2022). It is proposed that OXPHOS exerts dual roles in cancer progression. An imbalance in mitochondrial homeostasis results in excessive production of reactive oxygen species (ROS), which subsequently causes DNA damage, apoptosis, aging, and promotes cancer progression (Lin et al., 2022). Interestingly, a study that performed a comprehensive molecular characterization of mitochondrial genomes across various human cancers also found that genes such as ND2 and COX1 were frequently mutated in multiple cancer types (Yuan et al., 2020). Another study suggested that alterations in mtDNA coding genes might be associated with the development of EC, which further supported our findings (Liu et al., 2017).

Heteroplasmy is a distinctive feature of the mitochondrial genome and a typical feature of pathogenicity. When the pathogenic threshold is exceeded, the level of heteroplasmy can influence the biochemical and clinical phenotype, ranging from mild functional impairment to the complete disassembly of the mitochondrial complex (Su et al., 2016). Our study found that nearly half of the mutations in all EC patients were heteroplasmic mutations. Additionally, the average number of heteroplasmic mutations in EC patients was significantly higher than that in the healthy control group. EC, characterized by substantial genetic heteroplasmy, may involve specific organ system, giving rise to a diverse array of clinical manifestations (Xiang et al., 2022). Tissues with high energy requirements are especially prone to energy deficiencies, making them the most commonly affected (McFarland et al., 2010). In recent years, numerous studies have progressively identified that the level of mtDNA heteroplasmy plays an important role in the onset of various diseases, suggesting its potential as a biomarker (Zhou et al., 2024; Hong et al., 2024; Sazonova et al., 2024; Calabrese et al., 2022). A study that investigated the distribution of mtDNA heteroplasmic mutations in keratoconus (KC) patients reported no significant differences in non-synonymous heteroplasmic or homoplasmic variants within protein-coding regions between KC cases and controls, which was inconsistent with our findings. This discrepancy may be attributable to differences in the disease phenotypes examined. Nonetheless, their results remain a valuable reference for our study (Xu et al., 2023). Furthermore, these findings corroborate our results.

mtDNA mutations give rise to diseases with a wide range of manifestations and varying degrees of severity (Chung et al., 2022). Pathogenic mutations in mtDNA can impair the electron transport chain (ETC.), leading to an accumulation of excessive electrons. This excess can activate cancer-related pathways, which in turn exacerbate respiratory deficiency by promoting further mutations (Schon et al., 2012; Bernardino Gomes et al., 2021). Interestingly, although tRNAs represent merely 10% of the mitochondrial genome’s coding capacity, they carry over 50% of all pathogenic variants. In contrast, the protein-coding region-which constitute approximately 70% of mtDNA-account for around 40% of disease-associated mutations. The two rRNAs contain only about 2% of these pathogenic mutations (Schon et al., 2012). Among the tRNA genes, we identified three potentially pathogenic variants, one of which had already been reported in association with disease. According to Mitomap database, the 7496_T>C variant has been identified as a mutation associated with hearing loss, and it was located in the tRNA-Ser gene (Tang et al., 2015). Similarly, one recent study has identified the variants 5601_C>T and 5813_T>C, which are predicted to disrupt the secondary structure of their corresponding tRNAs, strongly suggesting that they may be potentially pathogenic (Ding et al., 2023). In this study, the three potentially pathogenic variants identified-7496_T>C, 5771_A>G, and 5613_T>A-were found to correspond to tRNA-Ser, tRNA-Cys, and tRNA-Ala, respectively. Such point mutations in mitochondrial genome tRNAs may influence the efficiency of processing at the 5′and 3′ends, trigger epigenetic alterations from specific post-transcriptional modifications, impair the accuracy of tRNA aminoacylation and codon decoding during translation, and ultimately compromise mt-tRNA stability, thereby potentially contributing to the development of EC (Shaukat et al., 2021).

In the protein-coding region, 10 frameshift variations were detected, which may introduce premature stop-codons during protein synthesis, leading to loss-of-function or disassembly of the complex. Besides, 14 mutations were predicted as deleterious by 13 bioinformatic programs in this study. Among them, 15395_A>G has been detected in Leber’s hereditary optic neuropathy (Cai et al., 2008). These 14 potentially pathogenic variants were located in the ND4, ND5, CYTB, ATP6, ATP8, and COX3 gene regions. Mutations in these mitochondrial genes may contribute to the development and progression of EC through multiple mechanisms. Mutations in ND4 and ND5 can impair the function of Complex I, leading to oxidative stress and metabolic reprogramming, thereby promoting tumor cell proliferation and resistance to apoptosis (Sharma et al., 2011). CYTB mutations disrupt the efficiency of the electron transport chain, resulting in excessive production of reactive oxygen species (ROS), which in turn activates DNA damage and oncogenic signaling pathways (Zhao et al., 2019). ATP6 and ATP8 mutations compromise ATP synthase activity, causing energy metabolism imbalance and increasing reliance on anaerobic glycolysis, which supports tumor survival under hypoxic conditions (Moreno-Loshuertos et al., 2023; Grzybowska-Szatkowska et al., 2014). COX3 mutations impair the oxidative phosphorylation function of Complex IV, further exacerbating ROS accumulation and mitochondrial dysfunction (Lee et al., 2025). Collectively, these alterations may synergistically drive EC initiation, progression, and therapeutic resistance.

We found that haplogroup D4 and D4-specific SNPs (8414_C>T and 14668_C>T) were significantly associated with a reduced risk of EC. One research team has identified haplogroups D, D4a, and D5 as potential genetic susceptibility haplogroups for EC in two high-incidence regions of China-the Chaoshan area and the Taihang Mountain area-which stands in contrast to the findings of the present study (Li et al., 2011; Li et al., 2007). Moreover, numerous other studies have reported associations between haplogroup D and the risk of various diseases. For example, A study demonstrated a relationship between haplogroups D and F and individual resistance to lung cancer in a Han Chinese population from southwestern China (Zheng et al., 2012). Another study showed that haplogroup D appeared at a significantly higher frequency among patients with endometrial cancer than in controls, suggesting a potential link between haplogroup D and the disease in southwestern China (Xu et al., 2006). Lastly, we found no specific genetic relationship between populations in the Chaoshan area and those in northern Sichuan-both high-incidence areas for EC. This genetic difference between populations may help explain why haplogroup D4 has been identified as a high-risk haplogroup in the Chaoshan area, whereas in our study, it appears to be a protective haplogroup. Additionally, applying Y-STR molecular genetic markers, our previous study examined the genetic background of populations from different geographic locations within the high-incidence EC area and revealed a strong genetic relationship between the northern Sichuan EC population and high-risk individuals in Chaoshan areas. This discrepancy from the present study’s findings may stem from the fact that our earlier results were derived from analyses based on only 24 common Y-STR gene loci, a method that is comparatively less accurate than the complete mitochondrial genome analysis employed in the current research (Jia et al., 2024).

In this research, we successfully sequenced the complete mitochondrial genome in individuals with EC and explored its potential link to EC development. As far as we are aware, this represents the first such investigation conducted in northern Sichuan, a region known for its high incidence of EC in China. We presented the first complete mitochondrial genome map of 146 EC patients and 120 matched controls, identifying 1299 variants-171 of which are novel-and thereby filling a critical gap in the regional mtDNA variant database. Our analyses also revealed a greater number of mitochondrial mutations and higher levels of heteroplasmy in the EC cohort compared to healthy controls, implying that these genetic alterations and mitochondrial heteroplasmy could play a role in EC development. By integrating thirteen independent bioinformatic programs, we pinpointed 3 high-confidence pathogenic tRNA mutations (5613_T>A, 5771_A>G, 7496_T>C) and 14 potentially deleterious coding-region variants, offering a ready-to-use list of candidates for functional validation. Earlier research teams successively conducted similar studies in the Chaoshan and Taihang Mountain areas-two major high-incidence areas for EC in China-but they examined only the HVR1 region. Compared with the present study, this approach is clearly limited and far from adequate for comprehensively characterizing variation across the entire mitochondrial genome. Nevertheless, our whole-mitochondrial-genome approach increased variant resolution roughly ten-fold, establishing a high-resolution paradigm for mtDNA–phenotype association studies in high-incidence populations. Moreover, we demonstrated that haplogroup D4 and D4-specific SNPs (8414_C>T and 14668_C>T) were significantly associated with reduced EC risk, providing new maternal genetic markers for individual risk stratification and early screening. Finally, we revealed no specific genetic relationship between populations in the Chaoshan area and those in northern Sichuan. Our previous study conducted a population-genetic analysis of EC using 24 commonly employed Y-STR loci. While these 24 Y-STRs offer only a paternal broad-brush outline, whole-mitochondrial-genome sequencing furnishes the maternal lineage with a high-resolution panoramic view, conferring irreplaceable advantages in reconstructing population history, refining phylogenetic resolution, detecting selection, and linking variants to functional outcomes.

Our study has several limitations. First, the development of EC is influenced by both environmental factors (such as diet, smoking, and alcohol consumption) and genetic factors. Failure to consider the interplay between these factors may introduce bias into the results. Additionally, the patients included in this study were solely from the high-incidence region of northern Sichuan, which may limit the generalizability of the findings to other regions or populations. Methodologically, the present study relied primarily on MitoMap, MitoTip, and 13 additional algorithms for variant annotation; however, the training datasets of these tools were predominantly composed of European or globally mixed populations, and their predictive accuracy in East Asian cohorts has not been independently validated. Moreover, mitochondrial DNA copy number (mtDNA-CN) was not quantified, yet copy-number alterations themselves are associated with tumorigenic risk and could represent an important confounding variable. Lastly, the study lacks functional validation; therefore, functional studies are essential to elucidate the underlying mechanisms of this impact.

## Data Availability

The original datasets are available in a publicly accessible repository: The original contributions presented in the study are publicly available. This data can be found here: [https://www.ncbi.nlm.nih.gov/genbank/, accession numbers: PX366769-PX367034].
